# On Stramonium

**Published:** 1811-06

**Authors:** 


### *For the Medical and Physical Journal.*

### *On Stramonium.*

(Continued from P. 385.)

BOERHAAVE has recorded some similar instances of the peculiar kind of ebriety and mental aberration occasioned by Stramonium, as well as some particular uses to which it was applied by the sensual natives of the East; in his Hist. Plant. quæ in horto Acad. Lugduni. Bat. crescunt, &c. it is stated "hæc utuntur meretrices Javanenses, dum a primariis sunt conductæ, tum hanc plantam maritis suis dant et delirii species subsequitur, unde coram maritis lascivia utuntur, et si hæc planta in majori copia detur, stupidi manent, languent et tandum moriuntur. Hæc planta facit mirificè insanire et temulentiam conciliat; qui hanc plantam comederunt, oculis apertis respondent, sed nullius rei memores sunt, nec putant sibi ulla re interesse; hinc tali potu utuntur Indi principes ad suos rivales stupidos reddendos, adeoque imperio minus capaces tamen in vivis servant, ut populo tum possint ostendere."

These effects are frequently attempted to be produced by a potion composed of opium, stramonium, and hyoscyamus, it is so mixed as to have little taste or smell; when taken in considerable quantity, or often repeated, the narcotic effects are seldom cured; for though, in a few instances, emetics given immediately after it, with large doses of salt and vinegar, afforded some relief, the faculties of the mind remained permanently injured.

Every part of the plant seems to possess active properties; but the effects produced by the seeds are the most violent. Blancardus relates that a girl 18 months old swallowed some seeds of Stramonium which she accidentally found. In a short time she raved, striking every thing, biting, and tearing the bye-standers with her nails, treading on the feet of others, and foaming at the mouth: she presently died in horrid convulsions. A boy of 30 months, who also swallowed some of the seed at the same time, was restored in 24 hours, by exciting vomiting with olive oil and sugar; an enema was injected, and cordial stimulating medicines were afterwards given.

A case is mentioned by Kramer, in which a man swallowed some seeds of Stramonium and fennel mixed with sugar in spirits of wine. It occasioned violent delirium; the patient was cured by drinking vinegar in which pepper was infused.

Hein has stated a shocking case of two infants who, in consequence sequence of swallowing some seed of the Thorn-apple, were affected with somnolency, burning heat of body, fury, swelling of the abdomen, convulsions, *strangulatio faucium*, *hydrophobia*. These violent and threatening symptoms were relieved by oleaginous remedies, milk, acid drinks, and clysters.

In a fatal case where some seeds were found in the stomach of the patient, the vessels of the brains were turgid with blood, and extravasation had taken place.

The fifth volume of Medical Commentaries contains two cases of the poisonous effects of the seeds of the Thorn-apple, by Mr. Thomas Fowler. The first, a girl near six years of age, October 11th, 1777, swallowed, between twelve and one o'clock, three fourth parts of the seeds of a fresh, ripe, middle sized Thorn-apple. "About two o'clock in the afternoon, she began to look stupid, seemed to forget herself, and gave incoherent answers. At three o'clock they gave her some bread and milk, which she attempted to swallow, but could not; upon which they laid her down upon the bed, where she seemed to sleep for about half an hour; but, when she awaked, her belly, tongue, face, and eyes were obviously swelled, and the two latter were also very red. These last symptoms abated about six o'clock; but, from half past three till seven, she seemed to sit like a perfect idiot.

About seven o'clock she seemed to have a motion to stool, and passed a living lumbricus teres, 14 inches long, with a little water, but without any fæces, or relief of symptoms; for, soon after, she began to grow worse, biting a man's hand, sometimes crying out that she saw cats, dogs, and rabbits, at the top, sides, and middle of the room; at other times with great eagerness catching at imaginary objects with her hands, and declaring that she saw many people who were not present. She suffered a continuance of these symptoms with little variation, and totally without rest, from eight o'clock in the evening, till six o'clock in the morning, being all that time restrained in bed by force in a raving and maniacal state." She had taken at half past eight in the evening half an ounce of ipecacuan wine, and four grains of ipecacuan powder, without any sensible effect being produced. Towards morning she had a costive stool. About eight o'clock, she took a pint of milk, and became more composed, though not rational. At nine o'clock Mr. Fowler found her "a little come to herself, but still frequently incoherent, and looking rather stupid. Her pulse was about 100 strokes in a minute, and her breathing not difficult." He gave her a powder with four grains of calomel and eight of jalap, as an evacuant. In half an hour she vomited freely, immediately fell into a sound sleep, and did not wake till near one o'clock, when she was perfectly sensible and composed, only complaining of a pain in her head. The purgative operated well in the afternoon, and in three days she was free from all complaints.

Another child aged nine, swallowed, at the same time with the preceding patient, her play-fellow, one fourth part of the seeds of a Thorn-apple, but was not at all affected with either idiotism or mania. "About three o'clock in the afternoon, she found herself affected with considerable head-ach, and hiccup and swelling of her face; but these symptoms went off by the usual time of going to bed; nevertheless she had but a troublesome night, awaking three or four times with sickness and vomiting." In the morning she arose free from all symptoms except a slight head-ach; she took a purgative and was quite well in two days.

Mr. Fowler calculated that the eldest child swallowed a dram and a half of the seed, and the youngest only half a dram, to which circumstance he attributes the difference of symptoms in the two patients.

The following case, by Dr. Abraham Swaine, well illustrates the effects of the Thorn-apple. "Robert Bulmer, a man of a strong constitution, 69 years of age, and who had enjoyed a good state of health all his life, till about two years before, when he was afflicted with the gravel; in October 1746, being advised by a friend to take a decoction of the fruit of the common burdock, as a remedy for his disease, by mistake gathered the fruits of the Stramonium or Thorn-apple. After dividing three of these, each of which was as big as a small hen's egg, into two parts, he boiled them in a pint of milk, which, when a little cooled, he drank off about eight o'clock in the morning fasting; presently afterwards, he became vertiginous or giddy; and therefore rose from his chair to take the air, with an intention to pluck more fruit. In walking two or three hundred yards from his house, he staggered as if drunk, feared he should fall on his head, and that he was about to lose his senses; but had no sickness or inclination to vomit. As soon as he got home he went to bed, and complaining of an excessive dryness of his tongue and throat, a little water mixed with wine was given him; he also felt an odd sensation of dryness in, and violent girding across the thorax. In less than half an hour he began to faulter in his speech, became insensible, restless, and muttered frequently; in which condition I found him. His extremities and also the trunk of his body were cold. His pulse small and quick: he often raised himself on his knees, continually stretching out his arms, and employed his hands as if searching for something he wanted; his eyes were dull and heavy; after some time he became dumb and more quiet, had almost most no pulse; and, upon his being taken out of bed that it might be put into better order, his limbs were visibly paralytic. Although he changed postures a little, yet he remained stupid for six or seven hours; then he raged furiously, requiring two persons to hold him in bed, notwithstanding which he raised himself up, tossed greatly, and seemed to catch at the bye-standers with his hands, uttering incoherent sentences. At last he became sensible and more quiet, restless and delirious by turns; and about ten o'clock in the evening of the same day perfectly recovered. After taking a purgative, he slept well all night, and had several stools in the morning. For the space of fourteen hours he neither slept, vomited, nor discharged any thing by stool or urine, though he frequently passes urine at other times, being grievously afflicted with the gravel."* Edin. Essays and Obs. Phys. and Lit. vol. 2.

The potent qualities of the Thorn-apple being sufficiently attested, it was natural to expect that physicians would attempt to apply them in the cure of certain diseases in which remedies of the narcotic class were more especially indicated. Storck has given us the result of considerable experience in the effects of the extract of Stramonium, in mania, epilepsy, &c. The first patient to whom he gave it was a girl aged twelve years, who had been insane about two months; the remedies she had taken had all failed of success. She answered questions incoherently, and could not distinctly express the words she uttered. She was morose, disobedient, and could not be induced by either mild or rough treatment to act with propriety. Half a grain of the extract of Stramonium was given her night and morning, and she was directed to drink after it a little veal-broth, or tea. After taking the pill fourteen days no alteration appeared; but in the third week, she began to be less stubborn, answered questions more readily, and spoke distinctly. In two months, (the remedy being continued, and in the second month repeated three times a day) she began to reason correctly, and regained her memory and other faculties. From this instance Dr. Storck concluded that the extract of Stramonium may be given safely, for a long time, and with good effect.

* The effects of a decoction of the seeds of Stramonium are well described by Bergius, Materia Med. tom. I. p. 122. "Decoctum è capsulis iij seminalibus Daturæ, in lacte coctis, hora 8 a m, epotum, vertiginem subito fecit, subsequente aphonia, agrypnia, frequente blatteratione, extremitatibus truncoque corporis frigidis, pulsu exili, celeri. Hunc statum excepit paralysis apparens totius corporis per horas 7, quæ tandem in furorem abiit. Hora 10 vespertina ad se rediit æger, et tranquille dormivit per noctem.

The

The second case in which he tried it was a woman aged 40, who had been affected with vertigo for two years; her mind was disturbed and insanity soon followed. Being brought to the hospital, she became furious, rose from her bed in the night, and by her wild exclamations disturbed and alarmed the other patients, some of whom she attempted to drag from their beds by force. Half a grain of the extract of Stramonium was given her night and morning. The first day she was more quiet; but became as furious in the night as before. The third day the dose was increased to a grain night and morning; the symptoms were rendered milder, and she continued to mend. On the 8th day the dose was increased to a grain three times a day. This quantity was continued to the fourth week, at which time all the violent symptoms had subsided, the mental functions were restored, and she ate and slept well. Still the vertigo recurred frequently and suddenly, and sometimes so severe as to threaten apoplexy, which indeed actually ended her sufferings in the course of a few months: "Vires sensim diminuti sunt, demum supervenit vera apoplexia, et subita mors." On examination after death, the veins of the cerebrum were found varicose; the anterior part of the falciform sinus was ossified for a considerable space; the two anterior ventricles of the cerebrum were distended and filled with hydatids of various form and magnitude. All the other viscera were sound. The Doctor pronounces this an incurable case, observing, "Quis enim medicus, etiamsi morbi cognovisset veram causam, potuisset hydatides tollere, aut sinum osseum ad mollitiem naturalem reducere?"

Dr. Storck has related some other cases in which extract of Stramonium proved useful in epilepsy and convulsions. In one patient he increased the dose to five grains in the day.

In Murray's *Apparatus Medicaminum*, the extract of Stramonium is said to have cured mania, and to have been given with good effect to women *post puerperium delirantibus.** In the Stockholm hospital, out of fourteen cases of epilepsy and convulsions eight were reported to be cured by Stramonium, five received relief, and one obtained no relief. Bergius mentions a case of mania, and two of convulsions that were quieted by this remedy. Reef, a Swedish physician, has stated two maniacal cases in which it proved of service. Murray has also cited from Wedenberg, that four patients, three of them girls of delicate habits, were liberated from convulsions by the use of this remedy. The fourth, a servant girl at Stockholm, who was supposed to have a dæmon, but

* This is confirmed by the testimony of Bergius. *Mat. Med.*

whose whose malady consisted in convulsions and a perverse imagination, was completely restored by it. He also mentions two cases of chorea, that were cured by the same means, one of them a young man, began with four grains of the extract, and the dose was gradually encreased to twelve grains. The other patient was a girl in whom the disorder appeared to have been contracted from fright.

Some cases of mania are stated in Ludwig's *Adversaria Med. Pract.* by L. I. E. Greding, M. D. in which he gave the extract of Stramonium to considerable extent, increasing the dose from one grain to ʒss in 24 hours. The paroxysms were quieted, and sleep and perspiration followed the use of the remedy. Some of the patients continued it till they had taken from ten drams to two ounces, with no other unpleasant effects than lax bowels and tormina; none of the cases however were cured by it. Dissatisfied with this want of success, Dr. Greding concluded that the extract which had been made according to Dr. Storck's directions was not good. He therefore obtained some on which he could depend, and prepared some pills with 17 oz. of the fresh extract of Stramonium, and 3½ oz. of the powder of the misletoe. Of this mass he formed pills of one grain each, which he gave to thirty-two patients with various success. None of them could bear more than six of the pills in twenty-four hours, and in most of the patients three or four of the pills were sufficient. " Nulli autem plura pilularum exhibere potuisse; cum plerique, sumtis tribus vel quatuor ejusmodi pilulis, de capitis obnubilatione, oculorum hebetudine, siti insigniter auctis et sat copiosa salivatione conquererentur, quod a longe majore dosi extracti Vindobonensis in præcedentibus non observaram."

Upon summing up the result of 46 cases of mania and epilepsy, it appears that the extract of Stramonium may be safely given in small and frequent doses. In several of the cases it immediately induced tranquil sleep, though in some the sleep was disturbed. In most instances the organ of vision was much affected, *oculorum hebetudo* being noticed by various observers. It occurred in two-thirds of the patients who took the extract prepared by Dr. Greding; he states, " Hi omnes enim pilula grani unius pondere, præsertim matutino tempore, vix deglutita, oculorum hebetudine plus minus gravi et binas horas ad minimum durante, adfligebantur ita, ut plerique tantum de oculorum caligine, ac si, reticulo bombycino tecti essent; quidam de oculorum scintillatione rerumque oculis objectarum vacillatione; aliæ de visu, dupliciter quin et oblique res cernente, alius et quidem adolescens quindecim annorum de presbyopia satis grave; et et alius denique de solis, absentis licet, splendore, oculos graviter adficiente, conquererentur. Temporaria vero hæc oculorum ægritudo, intermisso Stramonii usu, statim quoque evanuit, ita, ut certo asseverare possim, neminem omnium postea oculorum quoddam incommodum retinuisse. Ludwig *Adversaria Med. Pract.*

In some instances the remedy occasioned a shedding of tears, in others *lippitudo;* in some the head was attacked with pain, and the faculties were stupified; some were affected with ebriety, many with vertigo, and some with swooning fits. The imagination, in some, was principally touched, in others every mental faculty; "immo mentis plenariam perturbationem diu post ejus usum et tres denique faciem aliquoties rubore suffusam expertos esse. Ex quibus omnibus tam peculiares Stramonii agendi vis in caput, quam etiam veritas vetustæ jam opinionis, mentem ejus usu perturbari posse, satis elucessit." Ludwig.

Some of the patients were affected with extreme thirst, and dryness of the fauces, whilst in others a great flow of saliva was excited. The appetite during the use of the Stramonium was generally good, and occasionally even keener than natural, though it was sometimes less. In several instances bilious vomiting was occasioned, and the bowels were frequently affected with diarrhœa, tormina, and flatus. The secretions of perspiration and urine were promoted during its use. It also had much influence in provoking the catamenia; in one case they occurred after having stopped for four years. In four women vehement hiccup was observed; and in two a sense of itching over the whole body. From all these particulars Ludwig concluded, that besides its stupifying qualities, Stramonium possesses a remarkable acrimony, and when taken for a long time, has a powerful effect on the solids as well as the fluids of the body, exciting the secretory organs, and producing spasm and debility: his words are— "Hi omnes autem recensiti effectus clare demonstrant, Stramonio, parva dosi ingesto, præter sopiendi vim, insignem acrimoniam inesse, quæ, ubi diu sumere perseveraris, humores potenter resolvere, eosdem que ad varia organa se et excretoria majore copia allicere, solidaque nostri corporis, tono eorum hac ratione debilitate, variis spasmis obnoxia reddere possit. Quam ob rationem quoque evacuantia, inter ipsum ejusdem remedii usu data, plus nocuisse, quam juvasse videntur." Notwithstanding these properties of Stramonium, it appeared to exert very little salutary influence over the diseases in which it was administered; out of forty-six cases, alluded to by this writer, only one was cured; seven were made worse; and in three instances, death appeared to have been hastened hastened by its use. The successful case was a severe attack of epilepsy and convulsions; the former was entirely removed by the extract of Stramonium, but the patient never remained free from convulsions.

Externally applied, it is said to repel milk from the breasts; bruised and employed as a poultice, it has been recommended in some cutaneous affections, in burns, scalds, inveterate ulcers, and inflammation. Mr. Sawrey, who has devoted great attention to diseases of the eye, has found benefit from the application of an infusion of Stramonium in deep seated inflammation of that organ. It occasions dilatation of the pupil, and gives little pain.

From the preceding observations it will appear that Stramonium possesses powerful narcotic qualities, which operate especially upon the brain and nervous system. It may be given with safety in small doses, but in larger quantity it produces fatal effects. The diseases in which it has principally been administered have been mania, epilepsy, and convulsions; but the benefit derived from its use has been so rare and equivocal, that it never acquired the confidence of physicians, and modern practitioners have altogether discarded its use in such cases. The instances indeed cited by Storck are in favour of the remedy; but we must bear in mind that the Baron wrote a book on the subject, and evinced a degree of confidence in the medicine which his own experience hardly warranted, and which subsequent experiments have contradicted. The complaints alleged to have been relieved and cured by the thorn-apple, we must also recollect, frequently resist the action of all known remedies, while they occasionally terminate without our being able to assign any legitimate cause for the event. Unquestionably cases of mania, epilepsy, or convulsions, may exist, in which medicines of the narcotic class may prove beneficial; but they are not yet sufficiently defined. Dr. Cullen has remarked, "I have no doubt that narcotics may be a remedy in certain cases of mania and epilepsy; but I have not, and I doubt if any other person has, learned to distinguish the cases to which such remedies are properly adapted. It is therefore that we find the other narcotics, as well as the Stramonium, to fail in the same hands in which they had in other cases seemed to succeed. It is this consideration that has occasioned my neglecting the use of Stramonium, and therefore prevented me speaking more precisely from my own experience on this subject."*

*(To be continued.)*

---

* Mat. Med. vol. ii. p. 282.

